# Three-dimensional DNA nanomachine biosensor coupled with CRISPR Cas12a cascade amplification for ultrasensitive detection of carcinoembryonic antigen

**DOI:** 10.1186/s12951-024-02535-z

**Published:** 2024-05-18

**Authors:** Shuo Yao, Yi Liu, Yukun Ding, Xuening Shi, Hang Li, Chao Zhao, Juan Wang

**Affiliations:** State Key Laboratory for Diagnosis and Treatment of Severe Zoonotic Infectious Diseases, Key Laboratory for Zoonosis Research of the Ministry of Education, School of Public Health, Jilin University, Changchun, 130021 China

**Keywords:** DNA rolling machine, CRISPR-Cas12a, CEA, Cascade amplification

## Abstract

The detection of carcinoembryonic antigen (CEA) holds significant importance in the early diagnosis of cancer. However, current methods are hindered by limited accessibility and specificity. This study proposes a rapid and convenient Cas12a-based assay for the direct detection of CEA in clinical serum samples, aiming to address these limitations. The protocol involves a rolling machine operation, followed by a 5-min Cas12a-mediated cleavage process. The assay demonstrates the capability to detect human serum with high anti-interference performance and a detection limit as low as 0.2 ng/mL. The entire testing procedure can be accomplished in 75 min without centrifugation steps, and successfully reduced the limit of detection of traditional DNA walking machine by 50 folds. Overall, the testing procedure can be easily implemented in clinical settings.

**Graphical Abstract:**

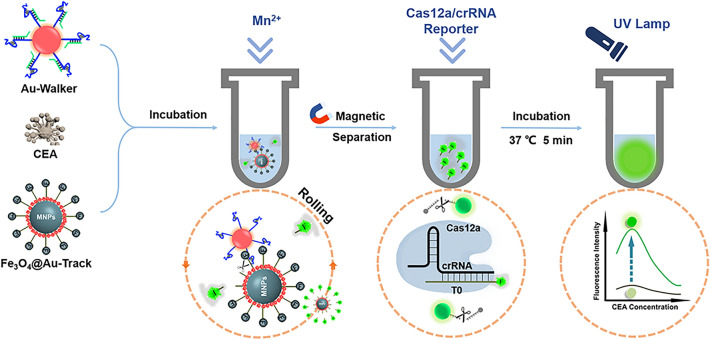

**Supplementary Information:**

The online version contains supplementary material available at 10.1186/s12951-024-02535-z.

## Introduction

Carcinoembryonic antigen (CEA) as a biomarker can be used as an indicator of various cancers [1]. The identification of CEA levels can aid in early cancer diagnosis or complement histopathological assessments. The concentrations of these protein biomarkers in serum, tissue, or saliva can be directly indicative of disease states. At present, CEA detection is widely achieved by using an enzyme-linked immunosorbent assay (ELISA) [2], which is relatively expensive and technically convoluted. Thus, the development of a method that is convenient, rapid, and economical for the selective detection of CEA and other protein biomarkers holds profound significance.

The clustered regularly interspaced short palindromic repeats (CRISPR)-associated proteins (CRISPR-Cas) system is a cutting-edge technology in gene editing and molecular diagnostics due to its unique characteristics, such as single CRISPR RNA (crRNA)-guided endonucleases that target and cleave DNA molecules [3]. The recent identification of non-selective collateral cleavage activities (trans-cleavage) on single-stranded DNA (ssDNA) has further expanded the capabilities of CRISPR systems in the field of nucleic acid analysis [4]. Recently, several amplification techniques have been incorporated into CRISPR-Cas12a detection systems to establish sensitive assays, such as polymerase chain reaction (PCR), recombinase polymerase amplification (RPA), loop-mediated isothermal amplification (LAMP), etc. [5]. However, the complexity of these amplification techniques poses a challenge as they are prone to false positives due to contamination. Furthermore, the widespread use of biosensing platforms employing CRISPR-Cas technology has been hindered by the lack of efficient methods to translate non-nucleic acid analyte recognition events into the trans-cleavage activity of CRISPR-Cas [6]. Despite the recent advancements achieved in non-nucleic acid analysis method, some current CRISPR-Cas related detection assays were based on immunoassay [7, 8], such as enzyme-linked immunosorbent assay (ELISA), although these methods achieve sensitive and versatile protein detection, they are still time-consuming, cumbersome, and not profit to on-site or primary-level laboratories detection. Some aptamer-assisted CRISPR-Cas assays have demonstrated the ability to rapidly detect small molecules or proteins [9, 10], however, these assays often require sophisticated electrochemical instruments or needs compromises between sensitivity and portability. Therefore, there is a critical need to develop a biomarker detection method that is user-friendly, does not dependent on complex instruments or equipment, and is suitable for on-site testing and point-of-care (POC) settings.

The DNA walking machine is a groundbreaking molecular device that falls within a unique category, demonstrating the ability to traverse predetermined one-, two-, or three-dimensional DNA tracks [11]. This technique has garnered widespread acceptance in various fields such as biosensing, drug delivery, and biological detection due to its impressive mechanical capabilities, precise orientation, and superior programmable control [12–14]. Of particular interest is the development of three-dimensional (3D) DNA walkers on nanoparticle surfaces, which has sparked significant enthusiasm in the realm of biological applications [15, 16]. Compared with the one-dimensional (1D) and two-dimensional (2D) nanomachines, 3D DNA walkers exhibit superior DNA loading capacity. This characteristic significantly achieved the enrichment of local track DNA, leading to enhanced walking efficiency and greater maneuverability [17]. This characteristic makes the 3D DNA walker an optimal tool for signal transmission and amplification. Nevertheless, the majority of 3D DNA walkers move incrementally, risking the walking leg to deviate from the track [18, 19]. Upon disengagement of the walking leg from the DNA track, the walking process abruptly terminated, leading to the cessation of signal amplification and a decrease in the sensitivity of 3D DNA walker-based sensors [20, 21]. A recent advancement in the field includes the development of a high-speed DNA rolling machine that travels along DNA or RNA tracks, driven by either a protein enzyme or DNAzyme [22]. Upon transitioning from walking to rolling motion, the walking process greatly improved, as the chance of derailment of leg DNA was largely reduced. In the realm of DNA rolling machines, it is common for such devices to utilize a 2D DNA track, involving the modification of DNA or substrate DNA on 2D nanomaterials in order to construct these machines. However, the use of a 3D DNA track can result in a higher concentration of track DNA, which can be advantageous for movement. Despite this potential benefit, the development of a 3D DNA rolling machine for the detection of CEA and other protein biomarkers has yet to be documented.

**Fig. 1:**
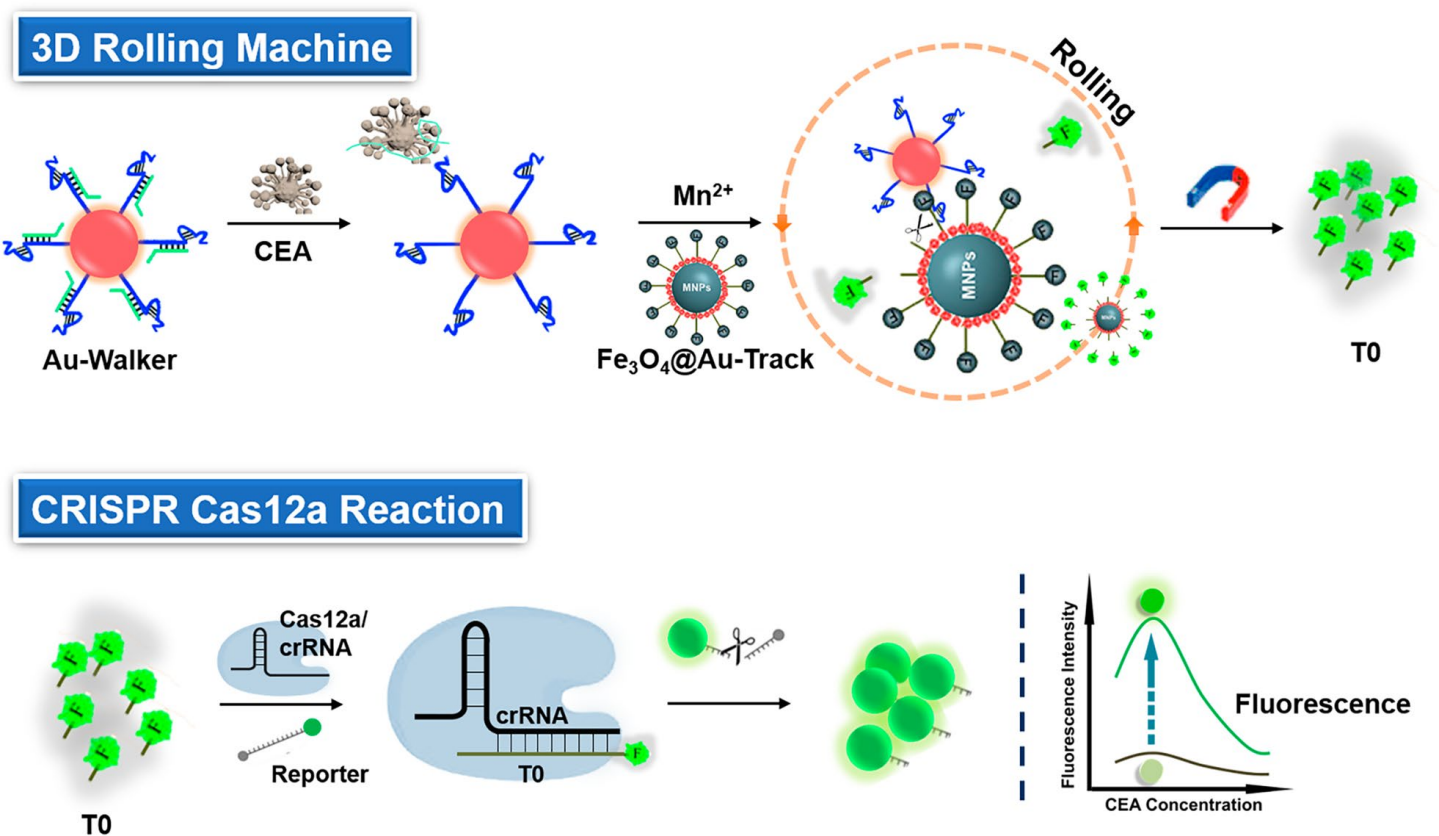
Schematic representation of CEA detection by 3D rolling machine and CRISPR-Cas12a

Here, we have devised a 3D DNA rolling machine that incorporates CRISPR-Cas12a amplification, providing a straightforward, visually accessible, and highly sensitive method for the direct detection of CEA (Fig. 1). Our method was developed using aptamer and AuNPs to efficiently convert and amplify signals. The aptamer with high affinity for CEA was used as a recognition element [23]. AuNPs were modified with aptamer-Walker DNA complex to create Au-Walker. Fe_3_O_4_@Au NPs were functionalized with Track DNA as Fe_3_O_4_@Au-Track. In the presence of CEA, the Walker strand was released, activating the 3D rolling machine to produce enzymatic fragments of Track DNA (T0). Following this, T0 acts as the target DNA to induce the cis- and trans-cleavage functions of CRISPR-Cas12a. Through the incorporation of the DNA rolling machine with CRISPR-Cas12a amplification, a notable improvement in analytical sensitivity was achieved. Demonstrating the feasibility of this innovative method, minute quantities of CEA were accurately identified and confirmed in clinical serum specimens. In conclusion, a novel instrument has been developed that allows for the convenient and highly sensitive detection of biomarkers.

## Experimental section

Materials and reagents, apparatus, synthesis of nanomaterials, optimization of conditions, asCas12a protein expression and purification, and Statistical analysis are described in the Supporting Information (SI).

### DNAzyme/Mn^2+^-assisted target cascade amplification

A mixture comprising 10 μL of Au-Walker, 30 μL of Fe_3_O_4_@Au-Track, and 40 μL of reaction buffer (containing 50 mM Tris–acetate and 200 mM NaCl, pH 8.0) was reacted with 10 μL of CEA at varying concentrations (0, 0.1, 0.2, 0.4, 0.5, 1, 5, 10, 20, 30, 40, 50, 60, 70, 80 ng/mL). After 20 min of incubation at 25 °C, 10 μL MnCl_2_ (15 mM) was added to initiate the DNAzyme and reacted at 25 °C for 50 min. After magnetic separation, 20 μL of supernatant was incubated with CRISPR-Cas12a reaction mix (including 3 μL as Cas12a enzyme (50 nM), 10 μL gRNA (50 nM), 1 μL ssDNA reporter (10 μM), and 6 μL 10 × NEB Buffer, added concentration) at 37 °C for 5 min. Finally, the fluorescence of the solutions was measured using excitation at 492 nm and emission at 518 nm.

### Protocol of rolling machine cascade amplification with real sample

The serum samples were obtained from human blood samples using standard separation procedures and stored at − 20 °C. Before detection, all serum samples were equilibrated to room temperature. Then, 10 μL of human serum samples were mixed with 10 μL of Au-Walker, 30 μL of Fe_3_O_4_@Au-Track, and 40 μL of reaction buffer and incubated at 25 °C for 20 min. Thereafter, 10 μL of 15 mM MnCl_2_ was added to initiate the DNAzyme for CRISPR-Cas12a reaction.

## Results and discussion

### The principle of CEA detection based on the rolling machine cascade amplification assay

To construct the rolling machine, a fraction of CEA aptamer was bound with walker DNA, forming a "DNA gate" that effectively secured the DNAzyme cleavage site. The DNA named Track DNA was modified to Fe_3_O_4_@Au NPs by the Au–S bond as the substrate of DNAzyme. Figure 1 illustrates the three stages of the cascade amplification assay, including (1) the activation of CEA-activated DNA walking on AuNPs; (2) the rolling of Au-walker on Fe_3_O_4_@Au-track surface and releasing DNA fragments (T0); (3) T0 activated the trans-cleavage of the ssDNA reporter by the Cas12a-crRNA complex. In the presence of the target, the CEA hybridizes with aptamer and releases Walker DNA, which then recognizes and digests the Track DNA by DNAzyme from the Walker. As the digestion of the track DNA progresses, the Walker DNA is released and forms a bond with an adjacent track. The presence of numerous walking DNA strands acting as legs on the surface of AuNP results in the Au-walker rolling instead of walking on the Fe_3_O_4_@Au NPs surface. This rapid rolling process releases a significant amount of target DNA of CRISPR-Cas12a to initiate the Rolling Machine-CRISPR/Cas12a Cascade Amplification Assay (Roller-Cas12a reaction), which can be visually observed or quantified using fluorescence detection methods. 

### Characterization of Au-walker and Fe_3_O_4_@Au-track

Morphological features of Au-Walker and Fe_3_O_4_@Au-Track were characterized by Transmission electron microscope (TEM), Dynamic Light Scattering (DLS), and Zeta potential investigations. To confirm the attachment of Walker DNA on the surface of AuNPs, we characterized Au-walker by TEM. Fig. S1A reveals that AuNPs were well dispersed with a uniform diameter of 15.3 ± 0.2 nm (by measuring the diameters of ~ 50 particles), respectively. After being decorated with the Walker and aptamer DNA, the size of AuNPs under TEM remained unaltered (Fig. S1B), whereas the hydrated particle size of Au-Walker increased from 21.0 ± 2.3 nm (Fig. S1C) to 68.1 ± 1.2 nm (Fig. S1D). According to Fig S1E, the UV–vis absorption peak of AuNPs at 520 nm was shifted to 525 nm, which illustrated that AuNPs are successfully decorated with DNA [24–26].

**Fig. 2:**
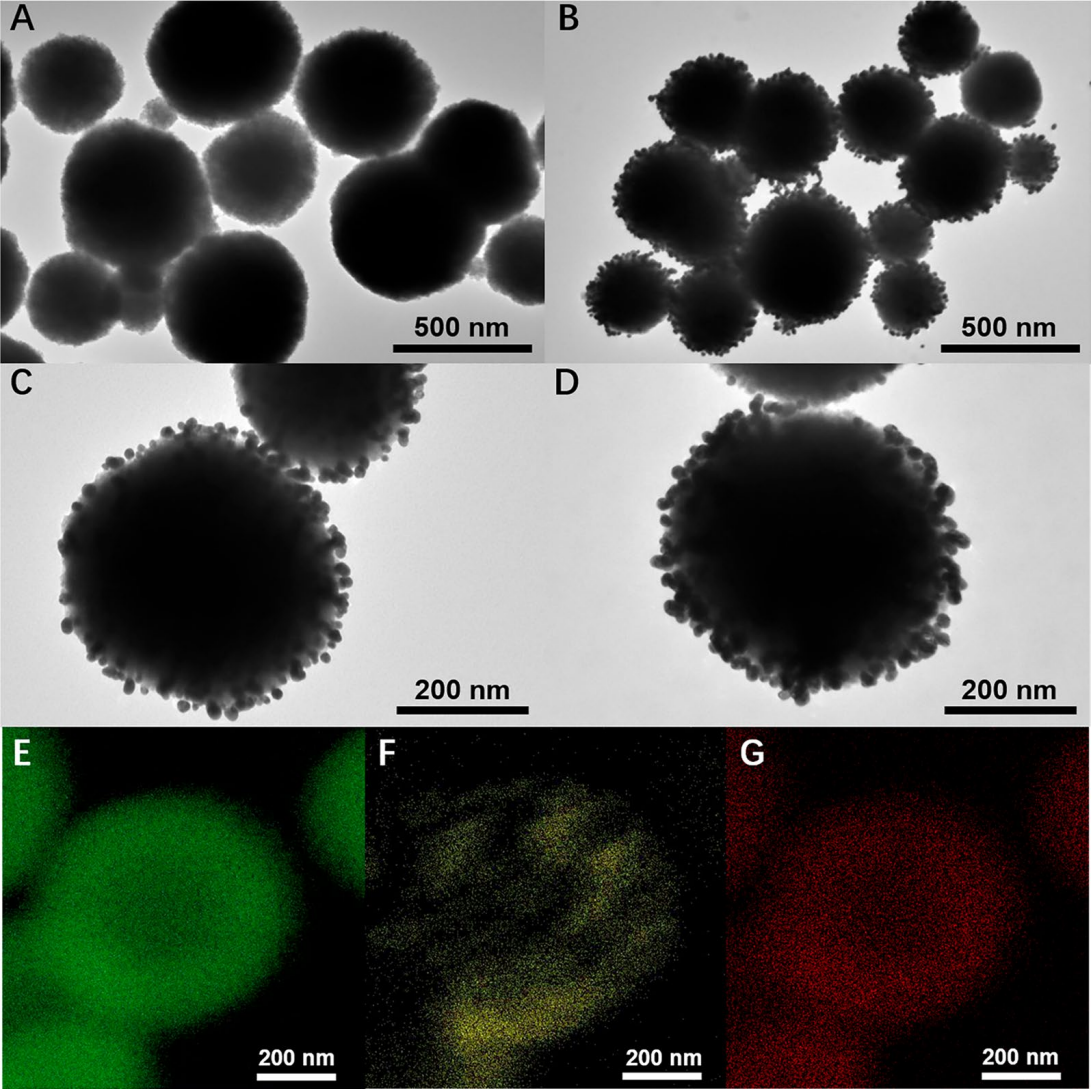
Characterization results of DNA rolling machine. TEM image of **A** Fe_3_O_4_ nanocores, **B** Fe_3_O_4_@Au NPs, **C** single Fe_3_O_4_@Au NPs, **D** Fe_3_O_4_@Au NPs-Track. TEM elemental mapping of Fe_3_O_4_@Au NPs-Track for **E** Fe, **F** Au, and **G** O

As shown in Fig. 2A, Fe_3_O_4_ nanocores were spherical, with an average size of 483.4 ± 33.0 nm. The prepared Fe_3_O_4_@Au nanoparticles are composed of Fe_3_O_4_ nanocores with gold seeds (Fig. 2B). The gold seeds (~ 15 nm) were uniformly and randomly attached to the surfaces of the Fe_3_O_4_ nanocores (Fig. 2C). Gold seeds appeared black, and Fe_3_O_4_ nanocores presented a light color in the TEM images, this can be attributed to the higher electron density of Au in comparison to that of Fe_3_O_4_. The TEM-mapping results further confirmed the coexistence of Fe_3_O_4_ and Au. After the modification of Track DNA, the morphology and size did not change significantly (Fig. 2D), the hydrated particle size increased from about 531.0 nm to 615.0 nm (Fig. S2B, C), which illustrated that Fe_3_O_4_@Au NPs were modified by DNA. Zeta potential results of Au-walker and Fe_3_O_4_@Au-Track were -38.4 ± 1.8 mV (Fig. S1F) and − 34.0 ± 0.08 mV (Fig. S2D), respectively. These results suggest that Au-walker and Fe_3_O_4_@Au-Track were synthesized and modified successfully and can be used for further experiments.

### Viability analysis

As previously stated, the Roller-Cas12a reaction involves two components: the CEA-mediated operation of the 3D rolling machine and the subsequent automated CRISPR-Cas12a reaction. The confirmation of walker release and DNAzyme cleavage of Track in the presence of Mn^2+^ was initially verified through agarose gel electrophoresis. According to Fig. S3, the presence of a band with a length of ~ 100 bp (Lane 3) corresponding to the hybridization of aptamer with walker. Due to the affinity of aptamer and CEA being greater than that of DNA complementary [27], the band at 100 bp became lighter (Lane 4) when the CEA was added, which indicates the CEA binds to the aptamer and dissociates the aptamer-walker complex. We further confirmed that the aptamer-walker complex had no DNAzyme activity that could not trigger the rolling process with Mn^2+^. As shown in Fig. S4, aptamer or aptamer-Walker complex and Track did not interact with each other in the absence of CEA (lane 6, 7, and 10), as no new band appeared. However, fast migration bands were observed after Track DNA was incubated with single Walker DNA and Mn^2+^ (lane 9), or aptamer-Walker complex, CEA, and Mn^2+^ (lane 11), indicating that it was effectively cut by the DNAzyme of Walker. These results demonstrate that the 3D rolling machine can only be activated in the presence of CEA and Mn^2+^.

**Fig. 3:**
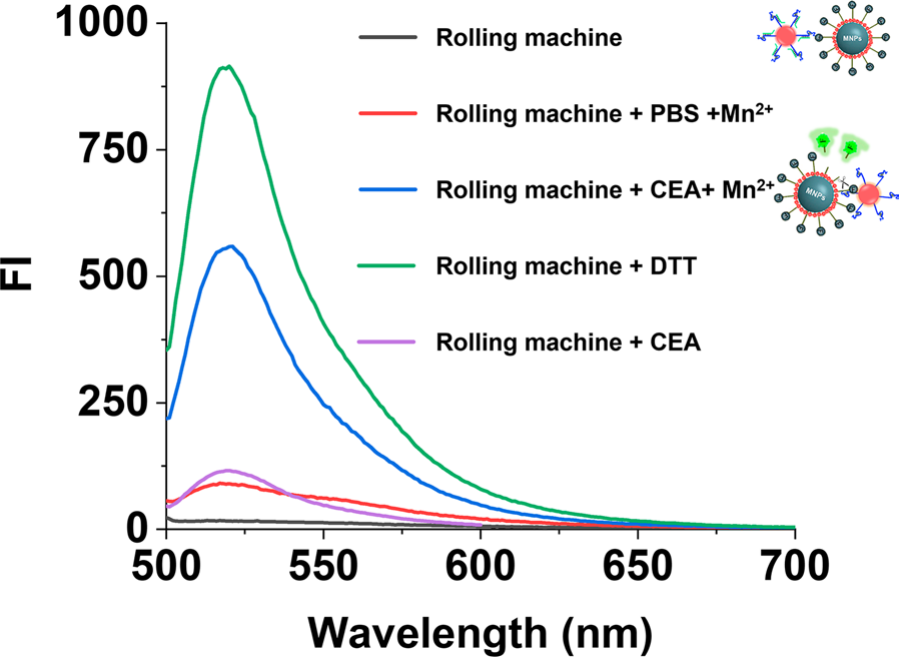
Viability analysis of DNA rolling machine. Fluorescence spectrum of rolling machine, rolling machine + PBS with Mn^2+^, rolling machine + 10 ng/mL CEA with Mn^2+^, rolling machine + 10 ng/mL CEA without Mn^2+^and rolling machine + DTT

The working principle was further interrogated through fluorescence analysis. Without adding CEA or Mn^2+^, the signal was negligible (Fig. 3, red and purple curve). Conversely, a significant rise in fluorescence signal was detected when the target was present (Fig. 3, blue curve). In addition, due to the modification of Track DNA on Fe_3_O_4_@Au through Au–S bonds, the DTT treatment was performed to reduce disulfide bonds. According to the green curve in Fig. 3, the strong fluorescence proves the successful preparation of the track components of the rolling machine. The above results demonstrated the expected viability of this strategy by showing that our Roller-Cas12a reaction was successfully activated exclusively in the presence of the CEA, which is consistent with Agarose gel electrophoresis.

Furthermore, to investigate the impact of nanoparticle characteristics on the rolling machine, we respectively modified DNA sequences on AuNPs and Fe_3_O_4_@Au NPs to form Au-Walker, Au-Track, Fe_3_O_4_@Au-Walker, and Fe_3_O_4_@Au-Track, and combine them in pairs to build the rolling machines. According to Fig. S5, in the presence of CEA, the Rolling machine I (Au-Walker and Fe_3_O_4_@Au-Track) and Rolling machine II (Au-Walker and Au-Track) exhibits obvious fluorescence intensity difference compared to PBS. However, rolling machine II requires centrifugation to obtain T0, which may hinder development of fast and convenient detection methods.

### Comparison between a DNA walking machine and a DNA rolling machine

According to previous work [28], increasing the local concentration of Track DNA effectively improves the walking efficiency of nanomachines, which can also improve signal output and equilibrium time. Therefore, we initially assessed the modification efficiency of the Track DNA in both the walking and rolling machines. An equal amount of Track was used for synthesizing the walking machine and rolling machine, and the prepared nanomachines were treated with DTT to reduce the Au–S bond, thereby releasing modified Track DNA. The ligation efficiency (LE) was calculated as follows:

$$\mathrm{Ligation\ efficiency}=\frac{F}{5{F}_{0}}\times 100\%$$

F0 represents the fluorescence intensity of the original Track DNA, while F represents the fluorescence intensity of the nanomachines treated with DTT. As shown in Fig. S6, the rolling mode exhibits higher ligation efficiency and releases more fluorescence intensity with CEA. The ligation efficiency of the rolling machine was 21%, compared to only 14% for the walking machine, indicating that our rolling machine has enhanced the local concentration of Track DNA.

**Fig. 4:**
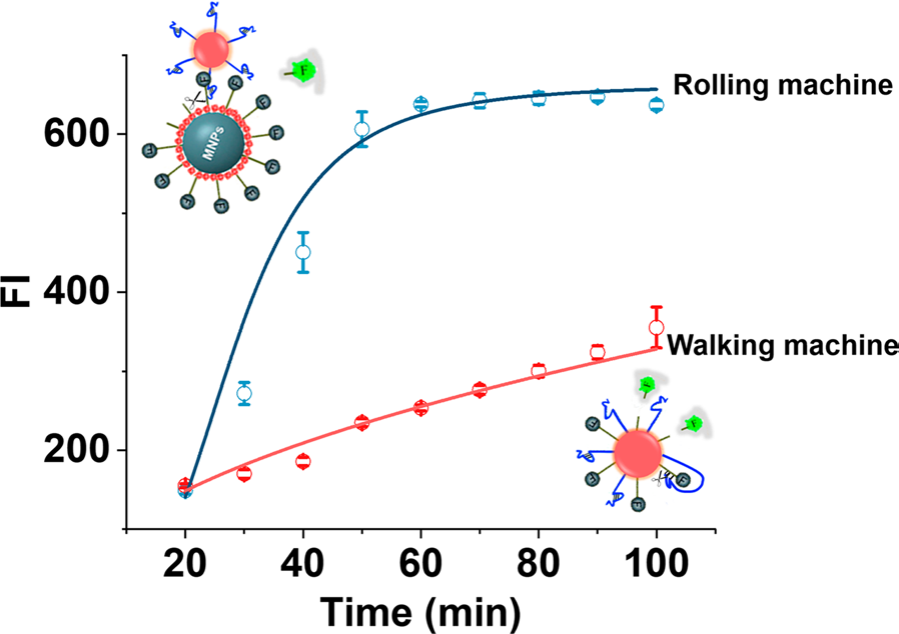
Kinetics comparison between rolling machine (blue curve) and walking (red curve). Consistent experimental parameters were applied to both the rolling and walking processes: Au-Walker (10 μL), Fe_3_O_4_@Au-Track (30 μL), Mn^2+^ (10 mM, 10 μL), and CEA (10 ng/mL, 10 μL). The concentration of Walker, aptamer, and Track conjugated with AuNPs was adjusted to an equivalent level (detailed procedure was described in SI)

The kinetics of the second-stage rolling process were compared with those of the traditional DNA walking machine, confirming the improved walking efficiency and signal amplification capabilities of the 3D DNA rolling machine. The Walker strand was initially attached to AuNPs utilizing the same experimental setup as that for the construction of Au-Walker to evaluate the rolling kinetics. The Au-Walker was then added in Fe_3_O_4_@Au-Track solutions along with Mn^2+^. The fluorescence signal of the supernatant was measured at different time intervals through magnetic separation. All experimental conditions for the walking mode were identical, except that an equal amount of walker strand and Track strand was modified on the same AuNPs to construct the traditional DNA walking machine. According to Fig. 4, our DNA rolling machine completed the reaction in only 50 min, compared to at least 100 min for a traditional DNA walking machine, effectively doubling the walking speed of the rolling machine relative to the walking machine. The highest fluorescence intensity of the rolling machine is twice that of the traditional walking machine, indicating that rolling nanomachines more readily release Track DNA. This could be because modifying Walker and Track DNA on different nanoparticles improves DNA binding and release, thereby demonstrating great potential for CEA detection.

### Optimization of the conditions

We optimized key experimental conditions to achieve optimal analytical performance, including the categories of Walker, the molar ratio of Au-Walker and Fe_3_O_4_@Au-Track, the concentration of Mn^2+^, the temperature, pH value, and reaction time. The impact of the aforementioned conditions on the change in fluorescence signals (F–F_0_) is depicted in Fig. S7, with detailed descriptions provided in the Supplementary Information (SI). Ultimately, medium binding length (9 nt) of Walker, 1:3 of Au-Walker to Fe_3_O_4_@Au-Track, pH 7.4, 10 μL 15 mM of Mn^2+^, incubation of 50 min at 25 ℃ were determined to be the optimal conditions and were utilized in subsequent experiments.

### Analytical performance

**Fig. 5:**
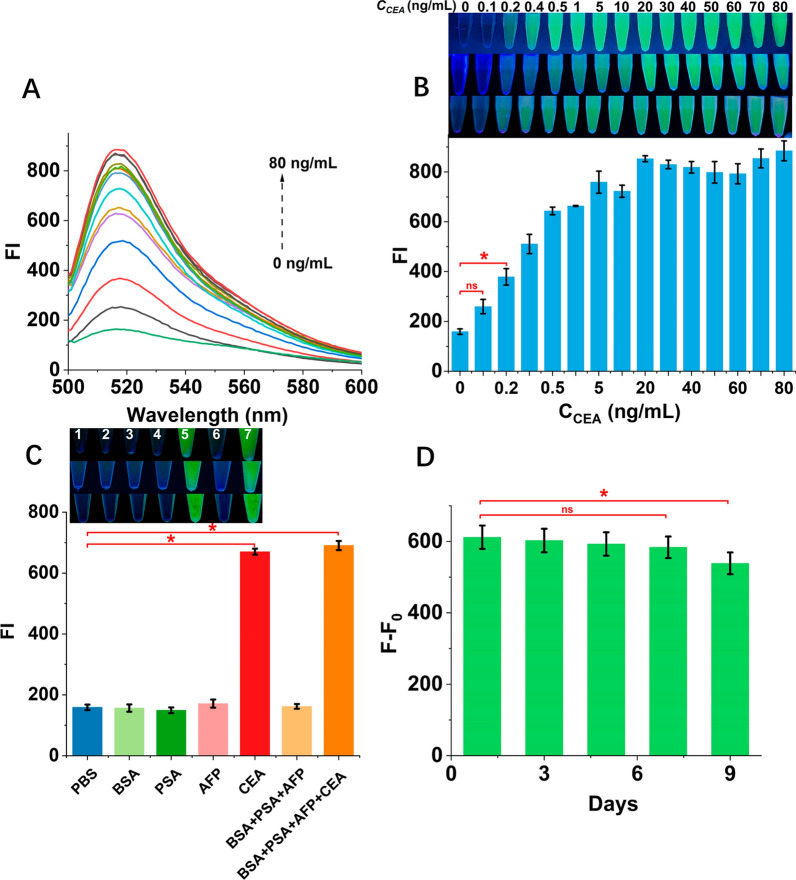
Sensitivity, selectivity, and stability test of Roller-Cas12a. **A** Fluorescence intensity responses of our assay for CEA at varying concentrations (bottom to top: 0, 0.1, 0.2, 0.4, 0.5, 1, 5, 10, 20, 30, 40, 50, 60, 70 and 80 ng/mL). **B** The fluorescence intensity versus the various concentrations of CEA. Insert: Images of the reaction system rolling machine combined with CRISPR-Cas12a. **C** Fluorescence intensity in the presence of different reagents (1 → 7: PBS, BSA, PSA, AFP, CEA, BSA + PSA + AFP, and BSA + PSA + AFP + CEA). The concentration of BSA, PSA, and AFP was each at 50 ng/mL. The concentration of CEA was 10 ng/mL and 10 mM PBS buffer was the blank control. **D** Stability of this assay within 9 days. F–F_0_ value of CEA at 10 ng/mL on 1, 3, 5, 7, and 9 days. Error bars represent the standard deviation of 3 replicates. *P < 0.05, “ns” means not significant (*P* > 0.05). Statistical analysis compares with 0 ng/mL (**B**), PBS (**C**), and 1st day (**D**), respectively

Under the optimal experimental conditions, the fluorescence intensity was recorded by a series concentration of CEA. In response to the absence of literature on the upper limit of serum CEA concentration, we extended the test range of CEA (0, 0.1, 0.2, 0.4, 0.5, 1, 5, 10, 20, 30, 40, 50, 60, 70 and 80 ng/mL) to accommodate clinical samples with abnormal concentrations. As demonstrated in Fig. 5A, the fluorescence intensity (FI) exhibited a gradual increase corresponding to the rising concentrations of CEA, ranging from 0.2 ng/mL to 20 ng/mL. When CEA concentration exceeds 20 ng/mL, FI reaches the plateau as CEA concentrations increase gradually. Green fluorescence was visible with a handheld UV lamp at concentrations as low as 0.2 ng/mL, establishing the detection limit of our assay at 0.2 ng/mL. Consequently, the limit of detection (LOD) for our visual assay was determined to be 0.2 ng/mL (the minimum concentration can be distinguished by naked eyes as criterion). Therefore, our assay indicated a “Yes” results when the concentration of CEA was higher than 0.2 ng/mL. In addition, the analytical performance of the traditional DNA walking machine integrated with CRISPR-Cas12a was evaluated. Based on Fig. S8, the DNA walking machine method exhibits a distinct fluorescent signal when CEA ≥ 10 ng/mL compared to blank controls. Therefore, the LOD of the walking machine coupled with CRISPR/Cas12a is 10 ng/mL (the minimum concentration can be distinguished by naked eyes as criterion). Our assay, based on the rolling machine, demonstrates high sensitivity and significantly reduces the LOD of the traditional DNA walking machine by 50-fold to 0.2 ng/mL, making it more suitable for developing methods for detecting CEA in clinical samples.

The specificity of the assay for CEA was further assessed by measuring bovine serum albumin (BSA), prostate-specific antigen (PSA), and alpha-fetoprotein (AFP). As depicted in Fig. 5C, BSA, PSA, and AFP exhibited fluorescence output at nearly background levels, underscoring the excellent selectivity of our assay.

Finally, to investigate the stability of the rolling machine combined with the CRISPR-Cas12a assay, three consecutive tests with 10 ng/mL CEA were conducted in parallel (Fig. 5D). Over a period of 9 days, this assay consistently exhibited a relative standard deviation (RSD) of 3.6%, demonstrating its good stability. These findings reveal that, in comparison to some earlier publications for biomarkers detection (Table S2), the rolling machine coupled with the CRISPR-Cas12a system has a similar detection limit and a shorter detection time.

### Detection of CEA in serum samples

To evaluate the practicability of the rolling machine combined with CRISPR-Cas12a assay in complex samples, 15 clinical human serum samples are analyzed, and the results were compared with those obtained using the Human CEA ELISA Kit (D711374, Sangon Biotech). According to previous reports, the concentration of CEA in the healthy human serum is less than 5 ng/mL [29, 30]. Therefore, a method that provides a simple Yes/No answer with a detection limit of 5 ng/mL is more advantageous for early tumor screening. In clinical sample testing, we optimized the concentration of gRNA (Fig. S8) to develop a straightforward, equipment-free, visual, and semi-quantitative assay suitable for on-site and point-of-care testing (POCT). The optimized is capable of producing "danger" or "safe" results based on the observable fluorescence intensity to the naked eye, enabling the differentiation between healthy individuals and those suspected of having tumors. As shown in Table 1, when the concentration of CEA is exceeds 5 ng/mL, our detection system shows strong fluorescence, indicating a potential tumor risk, and the diagnostic consistency between our assay and the ELISA kit was found to be 100%. When the concentration of CEA in human serum is approximately 5 ng/mL, the system shows weak fluorescence emission, suggesting a potential tumor presence. Expanding the scope of clinical screening in this way is advantageous for the early detection, diagnosis, and treatment of tumors. For lower serum CEA concentrations, our assay exhibits no fluorescence, indicating a "safe" status for tumor screening. For clinical serum samples with CEA concentrations below 5 ng/mL, the diagnostic consistency between our assay and the ELISA kit was 73%. Compared to the commercial ELISA kit, our assay features a shorter detection time (over 200 min for ELISA *vs.* 75 min for our assay) and is easy to operate. Additionally, compared to existing methods (Table S2), our assay evaluated clinical serum samples instead of spiked serum samples, offering better anti-interference capabilities and practicality.

**Table 1:** Comparison of commercial ELISA kit and our assay (n = 3, $\overline{x }\pm s$)

No	ELISA Kit (ng/mL)	This assay (Roller-Cas12a)		
		FI	Image	Output
1	1.12 ± 0.01	171.97 ± 4.32		Safety
2	1.20 ± 0.02	159.75 ± 3.16		Safety
3	3.87 ± 0.05	218.97 ± 6.42		Danger^a^
4	4.29 ± 0.01	267.74 ± 7.19		Danger^a^
5	1.43 ± 0.03	154.94 ± 3.71		Safety
6	0.39 ± 0.01	143.63 ± 2.53		Safety
7	0.43 ± 0.02	147.21 ± 2.42		Safety
8	0.99 ± 0.03	180.42 ± 4.20		Safety
9	0.22 ± 0.01	156.98 ± 6.80		Safety
10	1.02 ± 0.01	163.62 ± 7.45		Safety
11	15.11 ± 0.38	523.33 ± 11.40		Danger
12	17.42 ± 0.88	523.51 ± 8.17		Danger
13	4.55 ± 0.08	310.57 ± 6.42		Danger^a^
14	22.41 ± 1.50	601.79 ± 6.50		Danger
15	24.61 ± 1.26	603.27 ± 6.83		Danger

Safe: CEA < 5 ng/mL, no fluorescence by naked eyes

Danger: CEA > 5 ng/mL, obvious fluorescence by naked eyes

^a^ : Weak fluorescence but CEA < 5 ng/mL

## Conclusion

In summary, we have developed a user-friendly, rapid, sensitive, and specific assay using a rolling machine combined with CRISPR-Cas12a Cascade Amplification for the detection of CEA without a nucleic acid amplification step. Our method comprises a DNA rolling machine for target recognition, DNAzyme/Mn^2+^ cleavage of Fe_3_O_4_@Au-Track, and CRISPR-Cas12a amplification for signal output. The entire testing procedure can be accomplished within 75 min, achieving a LOD of 0.2 ng/mL for standard CEA. Our method demonstrates superior practical performance in measuring CEA in clinical samples compared to commercial ELISA kits. Significantly, this approach enhances test reliability by removing nucleic acid amplification and transcription processes, reducing the risk of false-positive and false-negative results. Furthermore, we hope to further optimize this assay to develop a quantitative system capable of detecting multiple tumor markers.

### Supplementary Information

Additional file 1 

## Data Availability

All data are available in the main text or the supplementary materials.
