# Supplementary material for: Three-dimensional DNA nanomachine biosensor coupled with CRISPR Cas12a cascade amplification for ultrasensitive detection of carcinoembryonic antigen
Source: J Nanobiotechnology. 2024 May 18;22:266. doi: 10.1186/s12951-024-02535-z (PMC11102226; doi:10.1186/s12951-024-02535-z)
Supplement: Supplementary file 1 — Additional file 1 [file 12951_2024_2535_MOESM1_ESM.docx]

**Supporting Information**

**Three-Dimensional DNA Nanomachine Biosensor coupled with CRISPR Cas12a Cascade Amplification for Ultrasensitive Detection of** **Carcinoembryonic antigen**

Shuo Yao, Yi Liu, Yukun Ding, Xuening Shi, Hang Li, Chao Zhao* and Juan Wang*

School of Public Health, Jilin University, Changchun 130021, China

* Email: czhao0529@jlu.edu.cn; [jwang0723@jlu.edu.cn](mailto:jwang0723@jlu.edu.cn)

**Contents**

[Tables 4](#_Toc153992173)

[Table S1. DNA sequences in this work 4](#_Toc153992174)

[Table S2. An overview for biomarkers detection 5](#_Toc153992175)

[Figures 5](#_Toc153992176)

[**Fig. S1** TEM results. 5](#_Toc132039406)

[**Fig. S2** DLS results. 6](#_Toc132039406)

[**Fig. S3** Agarose gel electrophoresis results. 7](#_Toc132039407)

[**Fig. S4** Agarose gel electrophoresis results. 8](#_Toc132039407)

[**Fig. S5** Fluorescence intensity of different Rolling machines. 9](#_Toc132039407)

[**Fig. S6** Ligation efficiency results. 9](#_Toc132039407)

[**Fig. S7** Optimization results 10](#_Toc132039408)

[**Fig. S8** Sensitivity test of DNA walking machine. 11](#_Toc132039409)

[**Fig. S9** Optimization of the concentration of gRNA. 11](#_Toc132039409)

[Materials and reagents 12](#_Toc153992177)

[Apparatus 12](#_Toc153992178)

[Synthesis of nanomaterials 13](#_Toc153992179)

[Synthesis of AuNPs 13](#_Toc153992180)

[Synthesis of Au-Walker 13](#_Toc153992181)

[Synthesis of Fe_3_O_4_@Au NPs 13](#_Toc153992182)

[Synthesis of Fe_3_O_4_@Au-Track 15](#_Toc153992183)

[Synthesis of Au-walking machine 15](#_Toc153992184)

[Synthesis of different rolling machine 16](#_Toc153992185)

[Fluorescence intensity measurement of different Rolling machines 16](#_Toc153992186)

[Agarose gel electrophoresis 17](#_Toc153992187)

[Viability analysis 17](#_Toc153992188)

[Optimization of the conditions 18](#_Toc153992189)

[Detailed procedures of optimization 18](#_Toc153992190)

[Results of optimization 19](#_Toc153992191)

[Stability test 20](#_Toc153992192)

[Optimization of the concentration of gRNA 21](#_Toc153992192)

[AsCas12a protein expression and purification 21](#_Toc153992193)

[Procedure of commercial CEA ELISA kit 21](#_Toc153992194)

[Statistical analysis 22](#_Toc153992195)

[Reference 22](#_Toc153992196)

## Tables

#### Table S1. DNA sequences in this work

| **Nanoprobes** | **Sequence (5` - 3`)** |
| --- | --- |
| CEA aptamer | ATA CCA GCT TAT TCA ATT |
| Walker-3nt | TTTTTTTTTTTTTTTTTTTTTTTTTTTTTTTTTTTTTTTTTTTAG AAC CGA ATT TGT GTCT **TAT** C TCC G AG CCG GTC GAA A TAG T |
| Walker-5nt | TTTTTTTTTTTTTTTTTTTTTTTTTTTTTTTTTTTTTTTTTTTAG AAC CGA ATT TGTG T**GG TAT** C T CCG AGC CGG TCG AA A TAG T |
| Walker-7nt | TTTTTTTTTTTTTTTTTTTTTTTTTTTTTTTTTTTTTTTTTTTAG AAC CGA ATT TGT **CTG GTA T**C T CC G AGC CGG TCG AA A TAG T |
| Walker | TTTTTTTTTTTTTTTTTTTTTTTTTTTTTTTTTTTTTTTTTTTAG AAC CGA ATT T**AG CTG GTA T**C T CCG AGC CGG TCG AA A TAG T |
| Walker-11nt | TTTTTTTTTTTTTTTTTTTTTTTTTTTTTTTTTTTTTTTTTTTAG AAC CGA AT **TA AGC TGG TAT**C T CCG AGC CGG TCG AA A TAG T |
| Walker-13nt | TTTTTTTTTTTTTTTTTTTTTTTTTTTTTTTTTTTTTTTTTTTAG AAC CGA **AAT AAG CTG GTA T** C T CCG AGC CGG TCG AA A TAG T |
| Walker-15nt | TTTTTTTTTTTTTTTTTTTTTTTTTTTTTTTTTTTTTTTTTTTAG AAC C **TG** **AAT AAG CTG GTA T**C T CCG AGC CGG TCG AA A TAG T |
| Track-3nt | TTTTTTTTTTTTTTCA CTAT /rA/G GAT AAG AT |
| Track | TTTTTTTTTTTTTTCA CCAT /rA/G GAT ACC AT |
| gRNA | AAUUUCUACUCUUGUAGAU CUGAUGGUCCAUGUCUGUU |
| Reporter | FAM-TTATT-BHQ1 |

Blue part: complementary part of Track and Walker

**Underline part:** complementary part of aptamer and Walker

#### Table S2. An overview for biomarkers detection

| **Methods** | **Targets** | **Time (min)** | **LOD** | **Samples** | **Ref.** |
| --- | --- | --- | --- | --- | --- |
| Electrochemistry | CEA | 75 | 0.84 pg/mL | Spiked serum | [1] |
| SPRi | CEA | 120 | 0.55 ng/mL | Spiked serum | [2] |
| ECIS | CEA | 90 | 14.9 fg/mL | Spiked serum | [3] |
| Photoelectrochemical | CEA | 120 | 0.3 pg/mL | Spiked serum | [4] |
| Biosensor | CEA | 90 | 0.1 ng/mL | Serum | [5] |
| Electrochemistry | HIV18 DNA | 60 | 1.95 pM | - | [6] |
| Electrochemistry | VEGF | 60 | 1.3 fM | - | [7] |
| Colorimetric | SARS-CoV-2 | 20 | 100 pM | - | [8] |
| Colorimetric | PSA | >300 | 0.1 ng/mL | Serum | [9] |
| Colorimetric | SARS-CoV-2 | 90 | 1 μg/mL | Plasmid | [10] |
| DNA Machine & CRISPR-Cas12a | CEA | 75 | 0.2 ng/mL | Serum | This assay |

## Figures

**
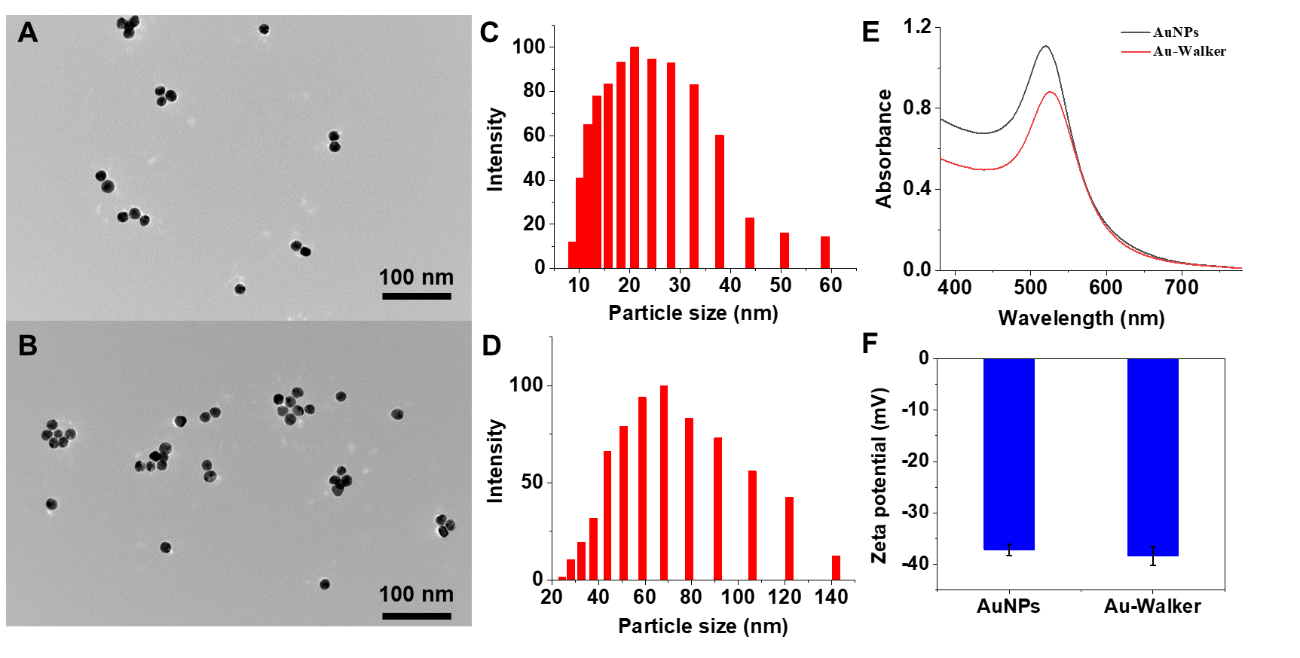
**

**Fig. S1** TEM image of (A) AuNPs and (B)Au-Walker. DLS results of (C) AuNPs and (D)Au-Walker. UV-vis absorption spectra of bare AuNPs (black) and Au- Walker (red). Zeta-potential of bare AuNPs and Au- Walker. Error bars represent the standard deviation of 3 replicates.

**
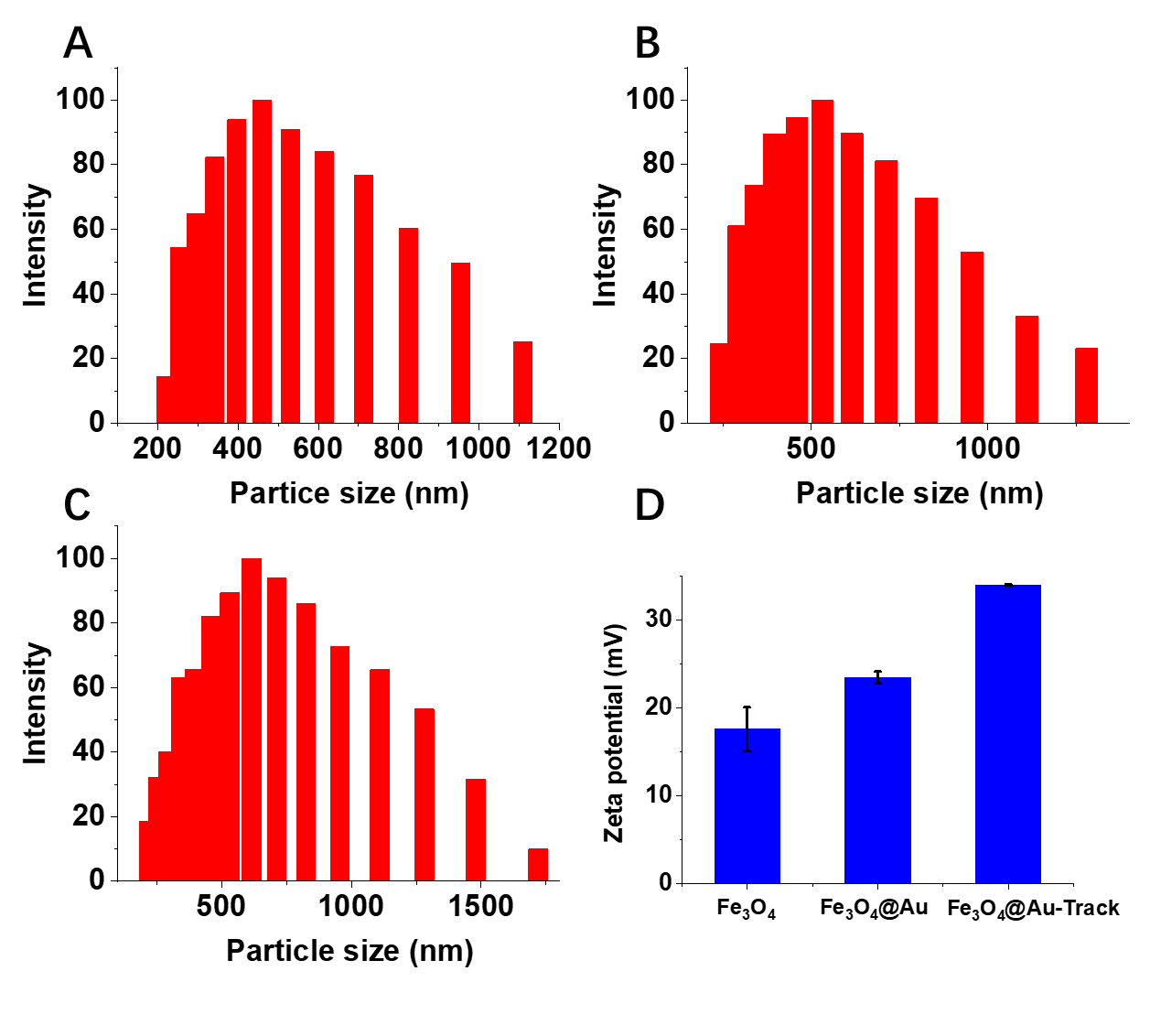
**

**Fig. S2** DLS results of (A) Fe_3_O_4_ nanocores, (B) Fe_3_O_4_@Au NPs and (C) Fe_3_O_4_@Au-Track. (D) Fe_3_O_4_ nanocores, Fe_3_O_4_@Au NPs and Fe_3_O_4_@Au-Track.

**
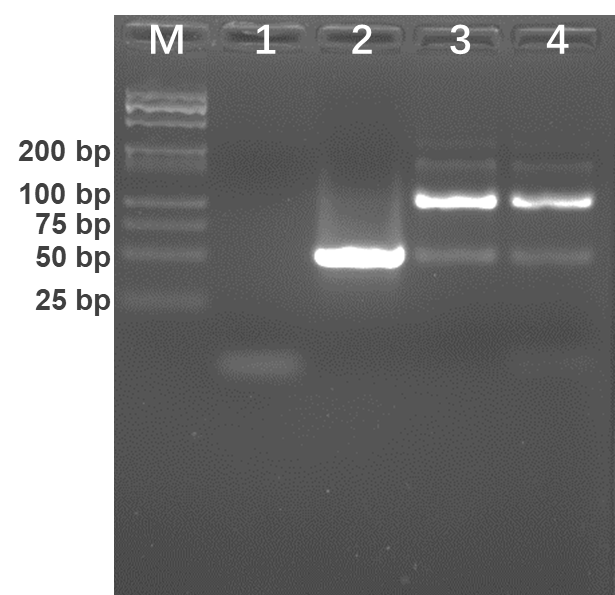
**

**Fig. S3** Agarose gel electrophoresis results. Lane M: marker, Lane 1: aptamer, Lane 2: Walker, Lane 3: aptamer-Walker complex, Lane 4: aptamer-Walker complex and 10 ng/mL CEA. The concentration of DNA strands was all 10 μM.


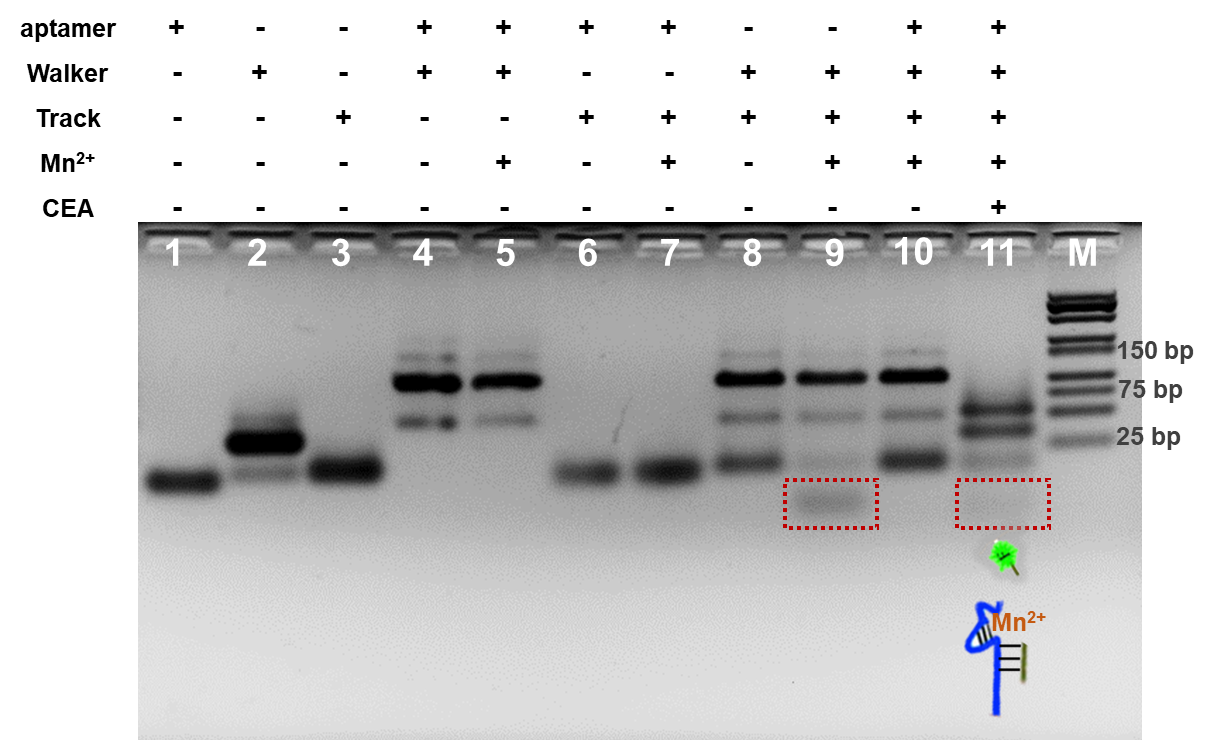


**Fig. S4** Agarose gel electrophoresis results. Lane M: marker, Lane 1: aptamer, Lane 2: Walker, Lane 3: Tack, Lane 4: aptamer-Walker complex, Lane 5: aptamer-Walker complex with Mn^2+^, Lane 6: aptamer and Tack, Lane 7: aptamer and Tack with Mn^2+^, Lane 8: Walker and Tack, Lane 9: Walker and Track with Mn^2+^, Lane 10: aptamer-Walker complex and Tack with Mn^2+^, Lane 11: aptamer-Walker complex, 10 ng/mL CEA and Tack with Mn^2+^.The concentration of DNA strands was all 10 μM.


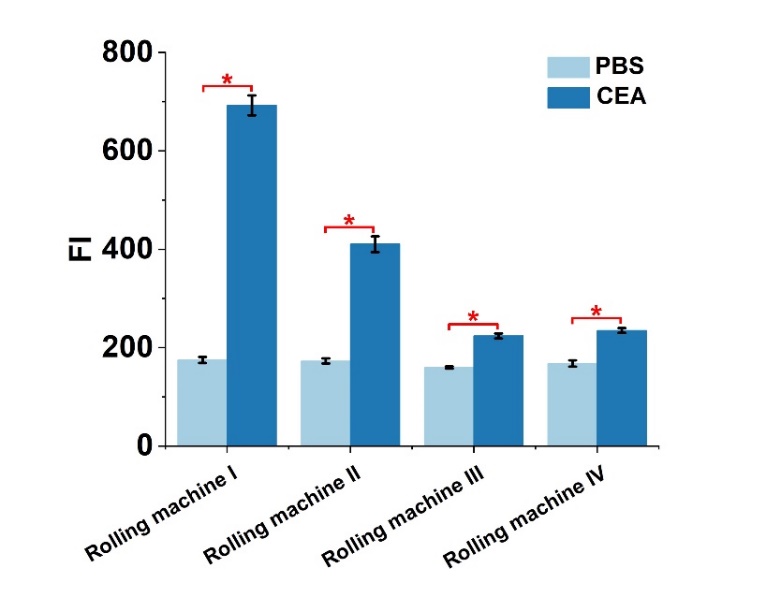


**Fig. S5** Fluorescence intensity of different Rolling machines. Rolling machine I: Au-Walker and Fe_3_O_4_@Au-Track, Rolling machine II: Au-Walker and Au-Track, Rolling machine III: Fe_3_O_4_@Au-Walker and Fe_3_O_4_@Au-Track, Rolling machine IV: Fe_3_O_4_@Au-Walker and Au-Track. The concentration of CEA was 20 ng/mL and dissolved in 0.01 M PBS. Error bars represent the standard deviation of 3 replicates. * *P*<0.05.


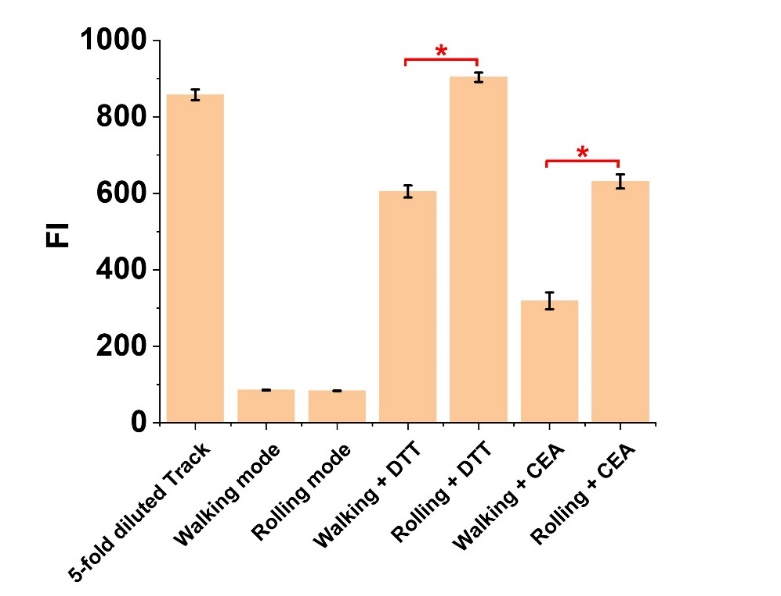


**Fig. S6** Ligation efficiency results. Fluorescence intensity of the 5-fold diluted Track DNA, rolling machine, walking machine, DTT treatment and incubation with CEA. DTT was 1 M and CEA was 20 ng/mL. Error bars represent the standard deviation of 3 replicates. * *P*<0.05.


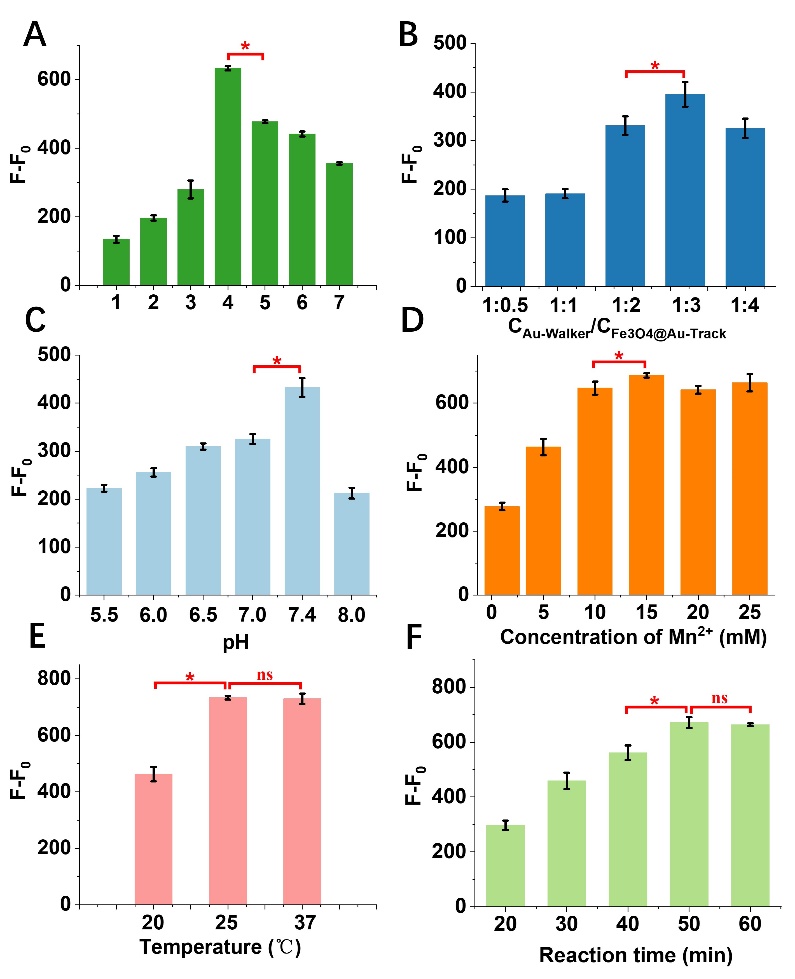


**Fig. S7** Optimization of (A) binding length of Walker, (B) the molar ratio of Au-Walker to Fe_3_O_4_@Au-Track, (C) value of pH, (D) the concentration of Mn^2+^, (E) the incubation temperature and (F) the reaction time. Error bars represent the standard deviation of 3 replicates. * *P*<0.05, “ns” means not significant.


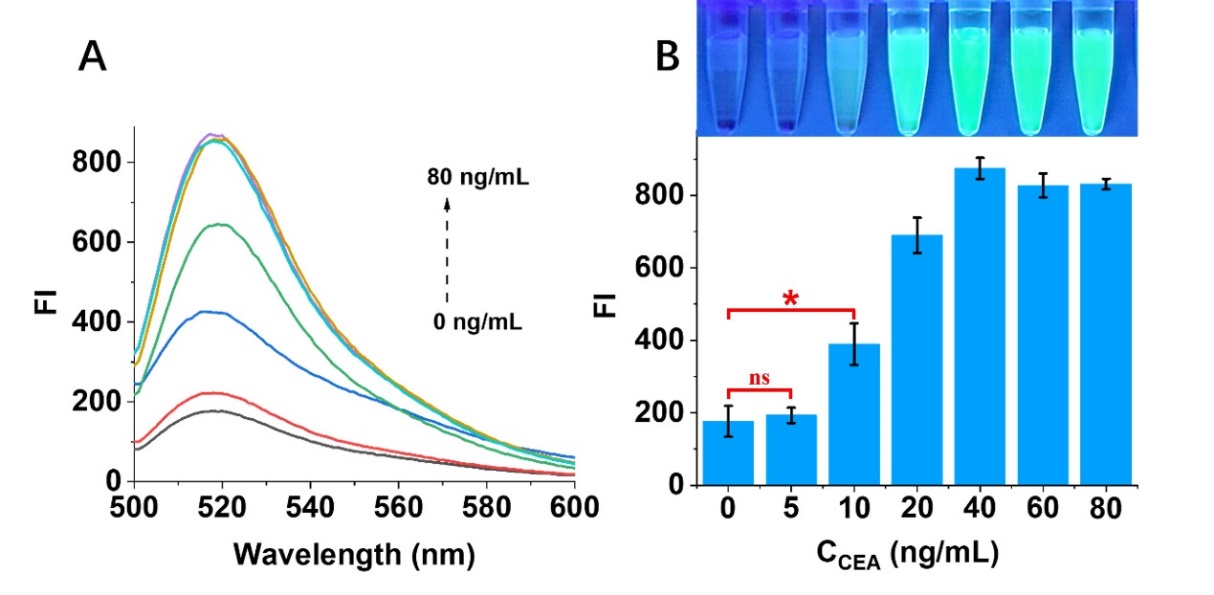


**Fig. S8** Sensitivity test of DNA walking machine coupled with CRISPR-Cas12a. (A) Fluorescence intensity responses of walking machine coupled with CRISPR-Cas12a for CEA at varying concentrations (0, 5, 10, 20, 40, 60 and 80 ng/mL from the bottom to the top). (B) The fluorescence intensity versus the concentration of CEA. Insert: Images of the reaction system walking machine coupled with CRISPR-Cas12a. * *P*<0.05, “ns” means not significant.

**
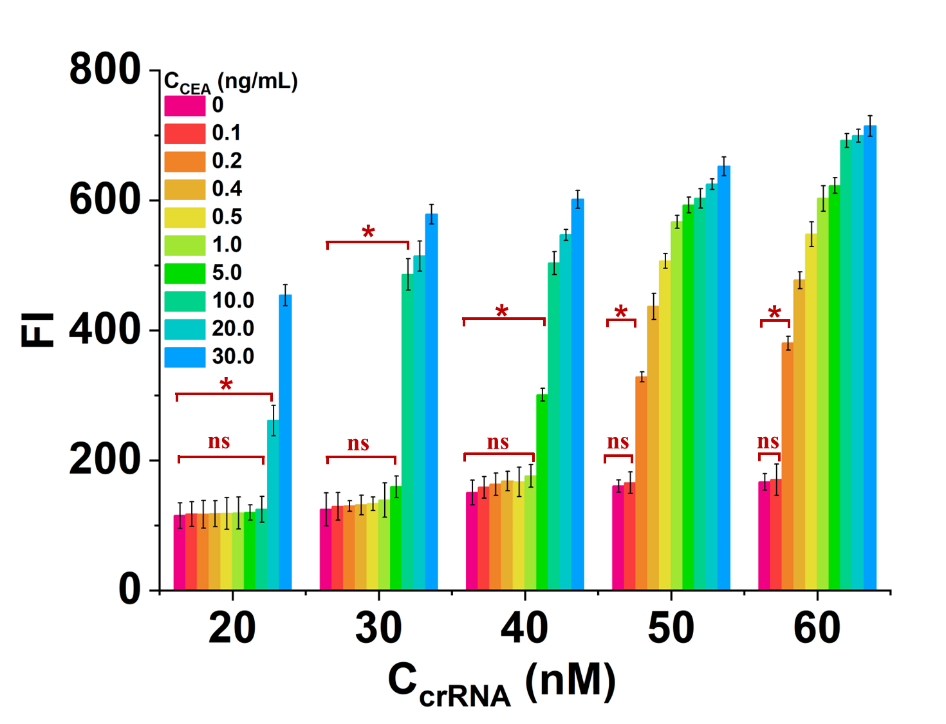
**

**Fig. S9** Optimization of the concentration of gRNA in CRISPR-Cas12a mix by 0, 0.1, 0.2, 0.4, 0.5, 1, 5, 10, 20, 30 ng/mL CEA. Error bars represent the standard deviation of 3 replicates. * *P*<0.05, “ns” means not significant.

## Materials and reagents

All oligonucleotides used in this study were synthesized and HPLC purified by Sangon Biotech Co., Ltd. (Shanghai, China). Sequences of the synthesized oligonucleotides are list in Supporting Information Table S1. Chloroauric acid (HAuCl_4_•3H_2_O), dithiothreitol (DTT), and Tris (2-carboxyethyl) phosphine (TCEP) were purchased from Sigma-Aldrich (http://www.sigmaaldrich.com). Hydrochloric acid (HCl), nitric acid (HNO_3_), sodium-citrate and citric acid were obtained from Beijing Chemical Reagent Co., Ltd, (Beijing, China). Phosphate-buffered saline (PBS, 0.01 M and 0.1 M, pH 7.4) was purchased from Sangon Biotechnology (Shanghai, China). CutSmart buffer (NEB buffer) was ordered from New England Biolabs, Inc. (Beverly, MA, USA). Methanol, polyethyleneimine (PEI, branched, MW ≈ 25,000 g/mol) and carbon disulfide (CS_2_), were purchased from Aladdin industrial Co., Ltd. (Shanghai, China). Iron chloride hydrate (FeCl_3_·6H_2_O) was purchased from Tianjin Guangfu Fine Chemical Research Institute (Tianjin, China). Ethylene glycol (EG), sodium dodecyl sulfate (SDS), sodium acetate (NaAc·3H_2_O), potassium hydroxide (KOH), were purchased from BEIJING DINGGUO CHANGSHENG biotechnology Co., Ltd. (Beijing, China). The aforementioned reagents were analytical grade and were utilized without further purification.

## Apparatus

The fluorescence intensity was measured with a spectrofluorophotometer (RF-5301PC, Shimadzu, Japan) using a 1 cm path-length quartz cell. Transmission electron microscopy (TEM) images of nanoparticles were performed on a JEM-2100F transmission electron microscope operated at an accelerating voltage of 200 kV (JEOL, Japan). The zeta potential measurements were determined using a NanoBrook 90Plus Zeta (Brookhaven Instruments Corp., USA).

## Synthesis of nanomaterials

### Synthesis of AuNPs

The well-dispersed AuNPs were directly synthesized via trisodium citrate reduction method described in our previous report[11]. All the glassware was cleaned with freshly prepared aqua regia (HCl/HNO_3_, 3:1, v/v) before using. Subsequently, 0.5 mL of HAuCl_4_•3H_2_O solution (1% w/v) in 52 mL of distilled water were heated to boiling with vigorously stirring, and then 2 mL of sodium-citrate solution (1% w/v, containing 0.05% w/v citric acid) were added into quickly. After further heating for 5 min, the mixture was cooled down to ambient temperature. The resultant citrate-stabilized AuNPs colloids were stored at 4 ℃ for further use.

### Synthesis of Au-Walker

Before thiolate oligonucleotides were mixed with the as-prepared AuNPs, they were incubated with TCEP at a molar ratio of 1:100 for 1 h to reduce the disulfide bond. For the preparation of walker-aptamer double strands, 15 μL of 10 μM walker strand was mixed with 15 μL of 30 μM aptamer in 1 × PBS buffer at 37 °C for 1 h. The use of excess of the aptamer was to ensure the complete inhibition of the formation of cleavage sites. After that, 1.5 mL of 2.5 nM gold nanoparticles were added. The mixture was incubated at -20 °C for 4 h, followed by thawing at room temperature. Finally, the solution was centrifuged at 12000 rpm for 20 min to separate the Au-Walker from the unreacted reagents. The Au-machine was washed 3 times with 10 mM PBS buffer (pH 7.4) containing 0.01% Tween 20 and was finally redispersed in 1 × PBS buffer.

### Synthesis of Fe_3_O_4_@Au NPs

*Synthesis of Fe_3_O_4_* *nanocore*

In a typical synthesis process for the Fe_3_O_4_ nanocore, 1.62 g FeCl_3_·6H_2_O were dispersed in 60 mL EG into a beaker with mechanical stirring at room temperature. After 30 min, 2.70 g NaAc·3H_2_O and 0.18 g SDS were added to the mixture. The mixture solution was vigorously stirred for 1 h until it became homogeneous. Then, the obtained homogeneous dark red solution was transferred to a 100 mL Teflon-lined stainless-steel autoclave and the autoclave was sealed and maintained at 200 °C for 18 h. After cooling to room temperature naturally, the products were obtained by centrifuging, sequentially rinsed with ethanol and deionized water for 3 times and subsequently dried under vacuum at 60 ◦C to obtain the Fe_3_O_4_ nanoparticles.

*Gold Seeds Synthesis*

20 mg trisodium citrate were dissolved in 2 mL deionized water. 1mL HAuCl_4_·3H_2_O (1 %, w/v) were diluted into 49 mL of deionized water with vigorous stirring. The mixture was subsequently heated under reflux at 105 ◦C and sodium citrates were added dropwise. The reaction mixtures were sustained for 15 min under stirring and reflux, leading to a buff to burgundy color change. The mixture was eventually cooled down to room temperature naturally.

*PEI-DTC Synthesis*

500 μL of 0.02 mmol PEI and 325 mg KOH were dissolved in 25 mL methanol under vigorously stirring with completely dissolved KOH. The solution was then purged with N_2_ for 15 s in order to exhaust the oxygen thoroughly, and 375 µL of CS_2_ was added by dripping slowly into the mixed solution. PEI-DTC was obtained with stirring for 10 min with a solution color change to light yellow.

*Fe_3_O_4_@ Au Nanoparticles Synthesis*

5 mg of Fe_3_O_4_ NPs were washed with methanol three times and 2 mL PEI-DTC solution was added. The mixture was then kept for 1 h. The precipitate was gathered with an external magnet and washed with deionized water three times and re-dispersed in 5 mL deionized water to prepare Fe_3_O_4_ coated PEI-DTC. Then 15 mL of gold seeds solutions was mixed with 5 mL Fe_3_O_4_ coated PEI-DTC at room temperature for 20 min to obtain Fe_3_O_4_@ Au NPs. The final products were washed with deionized water three times and stored at 4 ℃ for further use.

### Synthesis of Fe_3_O_4_@Au-Track

Track DNA was incubated with TCEP at a molar ratio of 1:100 for 1 h to reduce the disulfide bond. Then, 1 mg/mL Fe_3_O_4_@Au NPs was mixed with 60 µL of TCEP treated DNA (100 µM) and was incubated at room temperature for 16 h before aging. This process of addition of PBS buffer was repeated five times with a 1 h interval until the final concentrations of PBS buffer and NaCl were 10 mM and 300 mM, respectively. The solution was then incubated at room temperature for 24 h. After incubation, free track DNA was separated with an external magnet and washed 3 times with 10 mM PBS buffer (pH 7.4) containing 0.01% Tween 20 and was finally redispersed in 1 × PBS buffer.

### Synthesis of Au-walking machine

Before thiolate oligonucleotides were mixed with the as-prepared AuNPs, they were incubated with TCEP at a molar ratio of 1:100 for 1 h to reduce the disulfide bond. For the preparation of walker-aptamer double strands, 15 μL of 10 μM walker strand was mixed with 15 μL of 30 μM aptamer in 1 × PBS buffer at 37 °C for 1 h. The use of excess of the aptamer was to ensure the complete inhibition of the formation of cleavage sites. After that, 1.5 mL of 2.5 nM gold nanoparticles were added. The mixture was incubated at -20 °C for 4 h, followed by thawing at room temperature. After that, 60 µL of TCEP treated Track DNA (100 µM) and was incubated at room temperature for 16 h before aging. This process of addition of PBS buffer was repeated five times with a 1 h interval until the final concentrations of PBS buffer and NaCl were 10 mM and 300 mM, respectively. The solution was then incubated at room temperature for 24 h. After incubation, free track DNA was separated by centrifugation and washed 3 times with 10 mM PBS buffer (pH 7.4) containing 0.01% Tween 20 and was finally redispersed in 1 × PBS buffer.

### Synthesis of different rolling machine

To investigate the influence of nanoparticles features on the rolling machine, we respectively modified DNA sequences on AuNPs and Fe_3_O_4_@Au NPs to form Au-Walker, Au-Track, Fe_3_O_4_@Au-Walker and Fe_3_O_4_@Au-Track, and combine them in pairs to build the rolling machines, the details was as follows:

Rolling machine I: Au-Walker and Fe_3_O_4_@Au-Track, the same as the above protocols.

Rolling machine II: Au-Walker and Au-Track, the same as *Synthesis of Au-Walker*, for the synthesis of Au-Track, the difference is 1.5 mL of 2.5 nM gold nanoparticles was incubated with 60 µL of TCEP treated DNA (100 µM).

Rolling machine III: Fe_3_O_4_@Au-Walker and Fe_3_O_4_@Au-Track, 1 mg/mL Fe_3_O_4_@Au NPs was mixed with 15 μL of 10 μM walker strand, 15 μL of 30 μM aptamer to synthesis Fe_3_O_4_@Au-Walker, 1 mg/mL Fe_3_O_4_@Au NPs was mixed with 60 µL of TCEP treated DNA (100 µM) to synthesis Fe_3_O_4_@Au-Track.

Rolling machine IV: Fe_3_O_4_@Au-Walker and Au-Track, 1 mg/mL Fe_3_O_4_@Au NPs was mixed with 15 μL of 10 μM walker strand, 15 μL of 30 μM aptamer to synthesis Fe_3_O_4_@Au-Walker, 1.5 mL of 2.5 nM gold nanoparticles was incubated with 60 µL of TCEP treated DNA (100 µM) to synthesis Au-Walker.

## Fluorescence intensity measurement of different Rolling machines

Rolling machine I: A mixture comprising 10 μL of Au-Walker, 30 μL of Fe_3_O_4_@Au-Track, and 40 μL of reaction buffer (containing 50 mM Tris-acetate and 200 mM NaCl, pH 8.0) was reacted with 10 μL of CEA at 20 ng/mL. After 20 min of incubation at 25 °C, 10 μL MnCl_2_ (15 mM) was added to initiate the DNAzyme and reacted at 25 °C for 30 min.

Rolling machine II: 10 μL of Au-Walker, 30 μL of Au-Track, and 40 μL of reaction buffer (containing 50 mM Tris-acetate and 200 mM NaCl, pH 8.0) was reacted with 10 μL of CEA at 20 ng/mL. After 20 min of incubation at 25 °C, 10 μL MnCl_2_ (15 mM) was added to initiate the DNAzyme and reacted at 25 °C for 30 min.

Rolling machine III: 10 μL of Fe_3_O_4_@Au -Walker, 30 μL of Fe_3_O_4_@Au -Track, and 40 μL of reaction buffer (containing 50 mM Tris-acetate and 200 mM NaCl, pH 8.0) was reacted with 10 μL of CEA at 20 ng/mL. After 20 min of incubation at 25 °C, 10 μL MnCl_2_ (15 mM) was added to initiate the DNAzyme and reacted at 25 °C for 30 min.

Rolling machine IV: 10 μL of Fe_3_O_4_@Au -Walker, 30 μL of Au -Track, and 40 μL of reaction buffer (containing 50 mM Tris-acetate and 200 mM NaCl, pH 8.0) was reacted with 10 μL of CEA at 20 ng/mL. After 20 min of incubation at 25 °C, 10 μL MnCl_2_ (15 mM) was added to initiate the DNAzyme and reacted at 25 °C for 30 min.

## Agarose gel electrophoresis

Agarose gel (4%) was carried out to characterize different DNA strands and tests results. The final concentration of aptamer, walker and Track was all 10 μM. DNA strands were mixed and incubation at 25 °C for 20 min, followed by 10 μL MnCl_2_ (15 mM) was added to initiate the DNAzyme and reacted at 25 °C for 60 min. Each sample (10 μL) was mixed with 2 μL of 10 × loading buffer before loading onto the gel. Electrophoresis experiments were tested at 120 V for 30 min in 1 × TBE buffer. Subsequently, the gel was displayed *via* operating MINI Space 1000 (Tanon, China).

## Viability analysis

To further verify the feasibility of the experiment, fluorescence signal was detected with different substances in the detection system. For blue curve, 10 μL of CEA (20 ng/mL) was added in a mixture comprising 10 μL of Au-Walker, 20 μL of Fe_3_O_4_@Au-Track, and 50 μL of reaction buffer (containing 50 mM Tris-acetate and 200 mM NaCl, pH 8.0). After 20 min of incubation at 25 °C, 10 μL MnCl_2_ (10 mM) was added to initiate the DNAzyme and reacted at 25 °C for 30 min. After magnetic separation, 20 μL of supernatant was incubated with CRISPR-Cas12a reaction mix (including 3 μL as Cas12a enzyme (50 nM), 10 μL gRNA (50 nM), 1 μL ssDNA reporter (10 μM), and 6 μL 1× NEB Buffer) at 37 °C for 5 min. Finally, the fluorescence of the solutions was measured using excitation at 492 nm and emission at 518 nm. In addition, two control groups: Rolling machine and Mn^2+^ without the presence of CEA, and Rolling machine and CEA without the presence of Mn^2+^, was investigated to verify the feasibility. For negative group (red curve), the equal volume of PBS was introduced into the detection system to replace CEA, and the equal volume of DDW was introduced into the detection system to replace Mn^2+^. Ultimately, to prove that the fluorescence signal comes from rolling machine, 10 μL of Au-Walker, 20 μL of Fe_3_O_4_@Au-Track and 50 μL 1M DTT was incubated at 25 °C for 30 min to reduce disulfide bonds.

## Optimization of the conditions

### Detailed procedures of optimization

To achieve the best performance, several crucial experimental conditions were optimized. These include the kinds of Walker, molar ratio of Au-Walker and Fe_3_O_4_@Au-Track, the concentration of Mn^2+^, the temperature, pH and reaction time. The detailed procedures were as follows:

Based on the same steps, AU-walker were synthesized with various length of walker, listed in Table S1. 10 μL of different AU-walkers were mixture with 20 μL of Fe_3_O_4_@Au-Track and 50 μL of reaction buffer, respectively. After incubation with 10 μL of CEA (20 ng/mL) for 20 min, 10 μL MnCl_2_ (10 mM) was added and reacted at 25 °C for 60 min. After magnetic separation, 20 μL of supernatant was incubated with CRISPR-Cas12a reaction mix at 37 °C for 5 min and measured using excitation at 492 nm and emission at 518 nm.

For the optimization of the molar ratio of Au-Walker to Fe_3_O_4_@Au-Track, the volume of Fe_3_O_4_@Au-Track varied with a fixed Au-Walker volume of 10 μL, which is medium binding length (9 nt) of Walker. Reaction buffer serves as supplement to refine the whole volume for detection. The subsequent steps were consistent with the previous process.

For the optimization of pH, 10 μL of Au-Walker (9 nt) and 30 μL of Fe_3_O_4_@Au-Track were used for experiment. All the steps were the same as above, except for the value of pH, which was varied from 5.5 to 8.0, labeled in Fig.S5. To optimize the concentration of Mn^2+^, 0, 5, 10, 15, 20, 25 mM MnCl_2_ was used for detection, respectively. The rest steps were the same as above, except for pH, which had been optimized to 7.4. And the result of it was used for the next optimizations. For the optimization of temperature and reaction time, experiments were carried out at different temperature (20, 25, 37 °C) and rection time (20, 30, 40, 50, 60 min), respectively.

### Results of optimization

The walking efficiency might be related to the binding length of the Walker strand and aptamer, thus, we designed 7 Walker strands with different binding lengths and their performances are compared. As shown in Fig. S4A, the Au-Walker constructed with a Walker strand of medium binding length (9 nt) gives rise to the best performance. With a shorter binding length, aptamer was easy to dissociate from Walker strand, leaving high background fluorescence value. On the other hand, however, if the binding length are too long, they tend to hard to dissociate, preventing the operating of rolling machine. Then, it is shown in Fig. S4B that, with the increase of molar ratio of Au-Walker/Fe_3_O_4_@Au-Track, the fluorescence signal increases followed by decline as the molar ratio of Au-Walker/Fe_3_O_4_@Au-Track exceeds 1:3. The higher density of Au-Walker leads to a larger amount of walkable leg for the rolling machine, thus accelerating the rolling of AuNPs and improving the signal intensity. However, when the density of Au-Walker exceeds a threshold, it will be harder for DNAzyme/Mn^2+^ to access because of steric effect, leading to the reduction of signal amplification efficiency.

Moreover, due to the catalytic activity of DNAzyme was affected by the concentration of value of pH, Mn^2+^, temperature and reaction time, these experimental conditions were optimized. Due to the catalytic activity of DNAzyme was affected by pH, the pH values of buffer varied from 5.5-8.0 were investigated. According to Fig.S4C, The value of F-F_0_ reached the maximum at pH 7.4, and 7.4 was selected as the optimum pH. Metal ion was a critical cofactor affecting the catalytic activity of DNAzyme. Consequently, we investigated the influence of Mn^2+^ concentration and obtained that the catalytic activity of DNAzyme was most active in 15 mM Mn^2+^ (Fig. S4D). In addition, the reaction temperature and time were also optimized. As indicated in Fig. S4E, the optimum reaction temperature was selected as 25 ℃. The F-F_0_ value gradually increased with the increasing of reaction time until it reached a plateau when the reaction time was 50 min, shown in Fig. S4F. Thus, 50 min reaction time were used for subsequent experiments.

## Stability test

The stability of our assay was verified by detecting CEA at 10 ng/mL on 1, 3, 5, 7, 9 days. The procedure was the same as **DNAzyme/Mn^2+^-Assisted Target Cascade Amplification***.* Briefly, A mixture comprising 10 μL of Au-Walker, 30 μL of Fe_3_O_4_@Au-Track, and 40 μL of reaction buffer (containing 50 mM Tris-acetate and 200 mM NaCl, pH 8.0) was reacted with 10 μL of CEA (F) or 10 μL of PBS (F_0_). After 20 min of incubation at 25 °C, 10 μL MnCl_2_ (15 mM) was added to initiate the DNAzyme and reacted at 25 °C for 50 min. After magnetic separation, 20 μL of supernatant was incubated with CRISPR-Cas12a reaction mix (including 3 μL as Cas12a enzyme (50 nM), 10 μL gRNA (50 nM), 1 μL ssDNA reporter (10 μM), and 6 μL 1× NEB Buffer) at 37 °C for 5 min. Finally, the fluorescence of the solutions was measured using excitation at 492 nm and emission at 518 nm.

## Optimization of the concentration of gRNA

In clinical sample testing, we optimized the concentration of gRNA for clinical serum sample testing. For the optimization of gRNA concentration, 10 μL of Au-Walker (9 nt) and 30 μL of Fe_3_O_4_@Au-Track were used for experiment. All the steps were the same as **DNAzyme/Mn^2+^-Assisted Target Cascade Amplification***.* After magnetic separation, 20 μL of supernatant was incubated with CRISPR-Cas12a reaction mix (including 3 μL as Cas12a enzyme (50 nM), 1 μL ssDNA reporter (10 μM), and 6 μL 1× NEB Buffer) and 10 μL various concentration gRNA (20, 30, 40, 50, or 60 nM) at 37 °C for 5 min. Finally, the fluorescence of the solutions was measured using excitation at 492 nm and emission at 518 nm.

## AsCas12a protein expression and purification

The AsCas12a (Acidaminococcus sp. Cas12a) protein expression plasmid (gifted by Dr. Teng Fei) was transformed into E. coli BL21 (DE3) competent cells. When the culture reached 0.6 absorbance at OD600, 1mM Isopropyl β-D-thiogalactoside (IPTG) was used to induce protein expression at 18°C. After overnight induction, cell pellets were centrifuged and resuspended in lysis buffer followed by sonication. After gravity column purification, Glutathione Resin (GenScript, L00206) was used to pull down proteins. Glutathione, reduced (ChemCruz, sc-29094) was used to elute the protein. Amicon® Ultra-15 Centrifugal Filter Devices (Millipore, #UFC905008) was used to concentrate the protein, remove glutathione and exchange the storage buffer as well.

## Procedure of commercial CEA ELISA kit

(1) The number of the plates is pre-calculated, 30 min before experiment, take out the kit and restore it to room temperature. Then, add 100 μL standards and samples in each reaction well, the standard needs to be duplicated, incubated for 90 min at 37 °C.

(2) Discard the liquid and dry it. Add 100 μL/well biotin-conjugated antibody working solution to each reaction well, incubated for 60 min at 37 °C.

(3) Washing: Discard the liquid and dry it. Add 350 μL /well washing solution to each reaction well, and leave the washing solution after soak for 1~2 min. Repeat 4 times.

(4) Add 100 μL /well HRP-conjugated streptavidin working solution to each reaction well, incubated for 30 min at 37 °C.

(5) Washing: Add 300 μL/well washing solution to each reaction well, and leave the washing solution at intervals of 30 seconds. Repeat 4 times.

(6) Add 90 μL/well substrate reagent (hiding from light) to each reaction well, the color was developed at 37 °C for about 15 min.

(7) Add 50 μL/well stop solution to each reaction well, immediately measure the OD value at a wavelength of 450 nm with a microplate reader (within 5 min).

## Statistical analysis

All the data, except the special label, are presented as the means ± standard deviations ($\bar{x}\pm s$) of three parallel samples. Paired t-tests were conducted for statistical analysis using SPSS Statistics software (Version 25.0, IBM, USA). Statistical significance was set at 0.05.

## Reference

1. Niu C, Lin X, Jiang X, Guo F, Liu J, Liu X, Huang H, Huang Y: **An electrochemical aptasensor for highly sensitive detection of CEA based on exonuclease III and hybrid chain reaction dual signal amplification.** *Bioelectrochemistry* 2022, **143**.

2. Wu J, Zhang Q, Kang L, Wu X, Li D, Wang Y, Huang Y, Xue J: **Detection of carcinoembryonic antigens using a wavy gold-silver alloy nanoplate enhanced surface plasmon resonance imaging biosensor.** *Analytical Methods* 2022, **14:**4713-4720.

3. Tan Y-Y, Tan H-S, Liu M, Li S-S: **Electrochemical ratiometric dual-signal immunoassay for accurate detection of carcinoembryonic antigen in clinical serum based on rGO-Pd@Au-Thi and Chi-Fc-Au.** *Sensors and Actuators B: Chemical* 2023, **380:**133340.

4. Liu B, Ge Y, Lu Y, Huang Y, Zhang X, Yuan X: **An NIR light-responsive "on-off-on" photoelectrochemical aptasensor for carcinoembryonic antigen assay based on Y-shaped DNA.** *Biosensors & bioelectronics* 2023, **229:**115241-115241.

5. Li Y, Hu S, Chen C, Alifu N, Zhang X, Du J, Li C, Xu L, Wang L, Dong B: **Opal photonic crystal-enhanced upconversion turn-off fluorescent immunoassay for salivary CEA with oral cancer.** *Talanta* 2023, **258:**124435-124435.

6. Duan H, Wang Y, Tang S-Y, Xiao T-H, Goda K, Li M: **A CRISPR-Cas12a powered electrochemical sensor based on gold nanoparticles and MXene composite for enhanced nucleic acid detection.** *Sensors and Actuators B-Chemical* 2023, **380**.

7. Wang Y, Duan H, Yalikun Y, Cheng S, Li M: **A pendulum-type electrochemical aptamer-based sensor for continuous, real-time and stable detection of proteins.** *Talanta* 2024, **266**.

8. Liu S, Xie T, Pei X, Li S, He Y, Tong Y, Liu G: **CRISPR-Cas12a coupled with universal gold nanoparticle strand-displacement probe for rapid and sensitive visual SARS-CoV-2 detection.** *Sensors and Actuators B-Chemical* 2023, **377**.

9. Wang W, Liu J, Li X, Lin C, Wang X, Liu J, Ling L, Wang J: **CRISPR/Cas12a-based biosensor for colorimetric detection of serum prostate-specific antigen by taking nonenzymatic and isothermal amplification.** *Sensors and Actuators B-Chemical* 2022, **354**.

10. Zhang Y, Quan X, Li Y, Guo H, Kong F, Lu J, Teng L, Wang J, Wang D: **Visual detection of SARS-CoV-2 with a CRISPR/Cas12b-based platform.** *Talanta* 2023, **253**.

11. Fu K, Zheng Y, Li J, Liu Y, Pang B, Song X, Xu K, Wang J, Zhao C: **Colorimetric immunoassay for rapid detection of Vibrio parahaemolyticus based on Mn2+-mediate the assembly of gold nanoparticles.** *Journal of agricultural and food chemistry* 2018, **66:**9516-9521.
